# Fish can use coordinated fin motions to recapture their own vortex wake energy

**DOI:** 10.1098/rsos.231265

**Published:** 2024-01-03

**Authors:** Nils B. Tack, Kevin T. Du Clos, Brad J. Gemmell

**Affiliations:** Department of Integrative Biology, University of South Florida, 4202 East Fowler Avenue, Tampa, FL 33620, USA

**Keywords:** energy recapture, pectoral fin, particle image velocimetry, vortex interaction

## Abstract

During swimming, many fishes use pectoral fins for propulsion and, in the process, move substantial amounts of water rearward. However, the effect that this upstream wake has on the caudal fin remains largely unexplored. By coordinating motions of the caudal fin with the pectoral fins, fishes have the potential to create constructive flow interactions which may act to partially recapture the upstream energy lost in the pectoral fin wake. Using experimentally derived velocity and pressure fields for the silver mojarra (*Eucinostomus argenteus*), we show that pectoral–caudal fin (PCF) coordination enables the circulation and interception of pectoral fin wake vortices by the caudal fin. This acts to transfer energy to the caudal fin and enhance its hydrodynamic efficiency at swimming speeds where this behaviour occurs. We also find that mojarras commonly use PCF coordination in nature. The results offer new insights into the evolutionary drivers and behavioural plasticity of fish swimming as well as for developing more capable bioinspired underwater vehicles.

## 1. Introduction

Interactions between the bodies of swimming animals and the surrounding water act to influence their morphology and behaviour to enhance performance and lower the cost of transport [1–4]. While some swimmers, such as jellyfish, employ only a single control surface (i.e. the bell), most fishes can employ multiple fins collectively, which increases the possibility that flow interactions between control surfaces will occur [5–7].

The in-line arrangement of several fins, such as the dorso-ventral fins and the caudal fin, was shown to produce an optimal combination of flapping hydrofoils acting in tandem [8,9]. Vortex structures generated by the soft dorsal fin upstream can constructively interact with those produced by the caudal fin downstream to augment wake energy and enhance thrust [10–12]. Likely because of the historical emphasis on the wake left behind by undulating fish [6,13–17], the effects of the wake produced by the more anterior pectoral fins on downstream body structures have not been investigated. Also, perhaps because of the traditional classification of swimming modes and subgroups as discrete and independent gaits with distinct patterns of movements and propulsor use, the simultaneous display of two or more locomotor types is often regarded as a simple transitory behaviour rather than a distinct swimming mode [5,6,18]. Coincidentally, this has led to a gap in our knowledge about the prospect that the paired pectoral fins can move large amounts of water rearward towards the caudal fin, whose synchronized motions may enable the interception of upstream vortex structures.

This study explores how free-swimming silver mojarras (*E. argenteus*) coordinate their caudal fin and pectoral (paired) fins. We compare this gait with two commonly displayed swimming modes, body and/or caudal fin (BCF) propulsion that generates thrust via the trunk and tail, and median and/or paired fins (MPF) locomotion that relies only on other control surfaces such as the pectoral, dorsal and/or anal fins to generate thrust. Using fin kinematics and experimentally derived fluid velocity and pressure fields, we visualized the wake pattern of the pectoral fins of free-swimming mojarras to investigate the interactions with the caudal fin. Do upstream wake structures shed by the pectoral fins positively influence the fluid forces acting on the downstream caudal fin? We address this question in light of recent works emphasizing the role of constructive animal–fluid vortex interactions that enhance performance [19,20]. We provide direct evidence that body–vortex interactions due to coordinated motions of several fins enable wake energy recapture to enhance the propulsive efficiency of the tail. As such, we propose pectoral–caudal fin (PCF) coordination as a novel energy-recapture fish swimming gait.

## 2. Material and methods

### 2.1. Animals

We collected silver mojarras (*E. argenteus*) (*n* = 25; body length BL = 9.03 ± 0.9 cm) from the Tampa Bay area (W. Courtney Campbell Causeway, Tampa, FL, USA) and South Lido Key beach (Sarasota, FL, USA). The fish were housed at room temperature (21°C) in 38-litre aquaria. The fish were fasted for 24 h prior to experimentation and acclimated in the swim tunnel at swimming speeds *U* < 0.3 BL s^−1^ for four hours. All experiments were conducted in accordance with the laws of the State of Florida and under IACUC protocols (Permit no. IS00005965) approved by the University of South Florida (Tampa).

### 2.2. Flow visualization

We used a 5-litre Brett-type swim tunnel respirometer (Loligo Systems, Viborg, Denmark) to investigate swimming speeds ranging from 0.3 to 2.0 BL s^−1^. The working section of the swim tunnel was 30 × 7.5 × 7.5 cm^3^ (length × width × depth), and initial flow characteristics were assessed using particle image velocimetry (PIV) to ensure a uniform velocity profile. Individual fish naturally swam steadily at the centre of the test section against the flow.

We used high-speed two-dimensional PIV to compute velocity fields around the fish. Top-down recordings were acquired by a high-speed, high-resolution digital video camera (Fastcam Mini WX 100; Photron, Tokyo, Japan) at 1000 frames per second (2048 × 2048 pixels). Seeding particles (20 µm polyamide, Dantec dynamics, Skovlunde, Denmark) were illuminated by two opposing, overlapping horizontal laser sheets (808 nm, 3000–6000 mW, OptoEngine LLC, Midvale, UT, USA) to eliminate shadows on either side of the fish (electronic supplementary material, figure S1). Several videos were acquired for each swimming speed and for each fish but only one sequence was selected for fluid flow analysis (see electronic supplementary material, video selection criteria).

Fluid velocity fields were computed using the DaVis 8.3 software package (LaVision, Göttingen, Germany). Image pairs were analysed with three passes of overlapping interrogation windows (75%) with decreasing size from 96 × 96 pixels to 64 × 64 pixels, yielding 128 × 128 vectors per frame. Masking of the body of the fish before image interrogation confirmed the absence of surface artefacts in the PIV measurements.

The time-average free-stream water velocity across the test section upstream of the fish was subtracted to reveal vorticial flow structures. Vortex jet angle was measured as the mean angle of inclination of velocity vectors comprising the central region of jet flow within vortex rings relative to the swimming direction (see electronic supplementary material).

### 2.3. Pressure calculations

Velocity fields were input to the Queen 2.0 package for Matlab to compute pressure fields (code availability: http://dabirilab.com/software). The validation of this method against experimental and computational data found that this two-dimensional approach accurately estimates the patterns, timing, and magnitude of pressure fields around fish-like swimmers [21]. Because this algorithm is sensitive to integration domain size, the fish maintained a two-body-width clearance between the walls of the flume on either side and half a body length upstream and downstream. While precautions were taken to fulfil the experimental requirements to achieve reliable pressure field computations, it is difficult to obtain high-resolution fields to reduce the variance in the data. The data provide reasonable estimates of the pressure fields but because of the low sample count (PCF and BCF only occur consistently at different speeds, thus yielding only a few samples for direct comparison of the two gaits at the same speed), the variance could not be assessed reliably. A custom program in MATLAB R2020a automatically identified the outline of the fish to calculate peak pressure at the fish–fluid interface along the caudal fin. Note that the final pressure estimates are relative to a zero reference pressure (ambient pressure).

### 2.4. Forces and efficiency calculations

Forces were calculated per unit depth (because PIV data were two-dimensional) as the product of the length of each segment between points along the outline of the fish, the pressure along each segment, and the unit vector normal to each segment, giving units of Newtons per metre (see electronic supplementary material). Thrust and drag are the axial components of the forces in the swimming direction. The net thrust produced by the tail was computed from the caudal peduncle to the tip of the superior caudal lobe. The hydrodynamic efficiency ŋ of the tail is the ratio of the power produced by the tail in the swimming direction to the total power, where the latter is the sum of axial and lateral power [22]. The velocity and vorticity fields obtained in this study provide good estimates of the energy produced by the pectoral fins and how much transfers to the tail using pressure fields and their effects on the forces acting upon the tail. This study focuses on the hydrodynamic efficiency of the tail only rather than all the appendages to highlight the net effects of intercepting upstream vortices with axial appendages.

### 2.5. Field experiments

Field observations were conducted at South Lido Key beach (Sarasota, FL, USA). Top-down video sequences of freely swimming mojarras were recorded at 24 frames per second using a Nikon D5200 (Nikon, Tokyo, Japan). This frame rate provided sufficient temporal resolution to accurately resolve the instantaneous kinematics of the fish and enabled prolonged continuous filming with no limitation on the recording duration due to file size. The tidal flow speeds the fish were subjected to in their natural habitat were measured from the raw videos using ImageJ Fiji [23] by tracking drifting particles travelling in the same plane as the fish (see electronic supplementary material).

### 2.6. Kinematics measurements

Kinematics measurements were performed from scaled two-dimensional PIV and field videos using ImageJ Fiji. The complex three-dimensional motions and fin deformation (cupping) of the pectoral fins during MPF and PCF swimming could not be captured with the methods presented here, thus preventing the accurate calculation of the magnitude and direction of the forces produced by the pectoral fins. The amplitude (normalized to BL) and frequency (s^−1^) of the caudal and left and right pectoral fins were measured at the distal end of these structures and were averaged over three beat cycles. The angle of the pectoral fins relative to the fish body and the angle of the tail relative to the swimming direction were also recorded over time. The proportion of time displaying PCF in the swim tunnel at 0.3, 0.5 and 0.7 BL s^−1^ was quantified for 10 min of continuous swimming. The duration of the experiments was deemed appropriate after observing that the fish did not transition to other swimming modes or behaviour over time, such as during the flume acclimation phase that lasted upwards of three hours. PCF coordination was characterized by the synchronized kinematics of the out-of-phase pectoral fins and the caudal fin over three or more consecutive tail beats. Phases during which the animals repositioned themselves or briefly swam near the test chamber walls were considered non-PCF.

### 2.7. Statistics

All statistical tests were performed using MATLAB R2020a. ANOVA F-test was used to test for significant differences in kinematics parameters between fins and swimming speeds. Tukey's *post* *hoc* tests were used to determine which group means differed significantly. A one-way ANOVA was used to test for significant differences in adduction vortex jet angle across swimming speeds during PCF. Two-sample *t*-tests were performed to compare peak pressure at the tail, net tail thrust, and the hydrodynamic efficiency (ŋ) of the tail between PCF and BCF at 0.5 and 0.7 BL s^−1^. Differences were considered significant at *p*-values less than *α* = 0.05.

## 3. Results

### 3.1. Hydrodynamics of pectoral–caudal fin coordination

**Figure 1.**
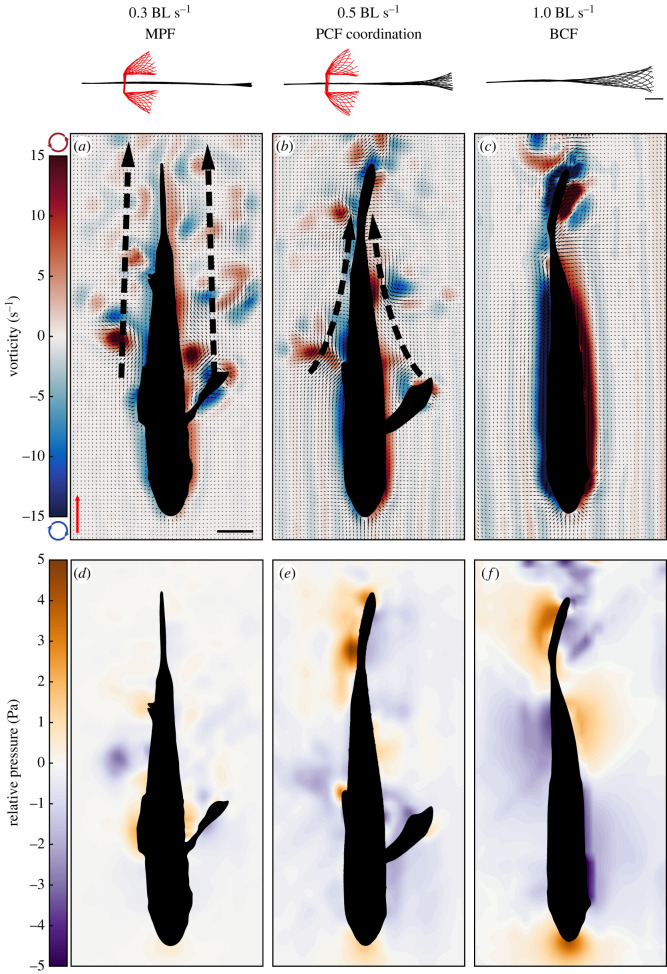
Instantaneous vorticity and pressure fields calculated for three speed-dependent gaits. (*a,d*) At 0.3 BL s^−1^, the fish relied on asynchronous median/paired fin (MPF) locomotion, did not engage the caudal fin (see fin and body centrelines at the top) and shed most of the pectoral fin wake in the free stream. (*b*) During PCF coordination at 0.5 BL s^−1^, pectoral fin vortices were circulated along the body where they interacted with the caudal fin, locally increasing pressure (*e*). Body-caudal fin (BCF) undulations produced no vortices upstream of the tail (*c,f*). The red scale arrow indicates 0.5 m s^−1^ and the scale bar indicates 10 mm for all frames and body–fin centrelines. Thick dashed arrows indicate the path of the pectoral vortices.

**Figure 2.**
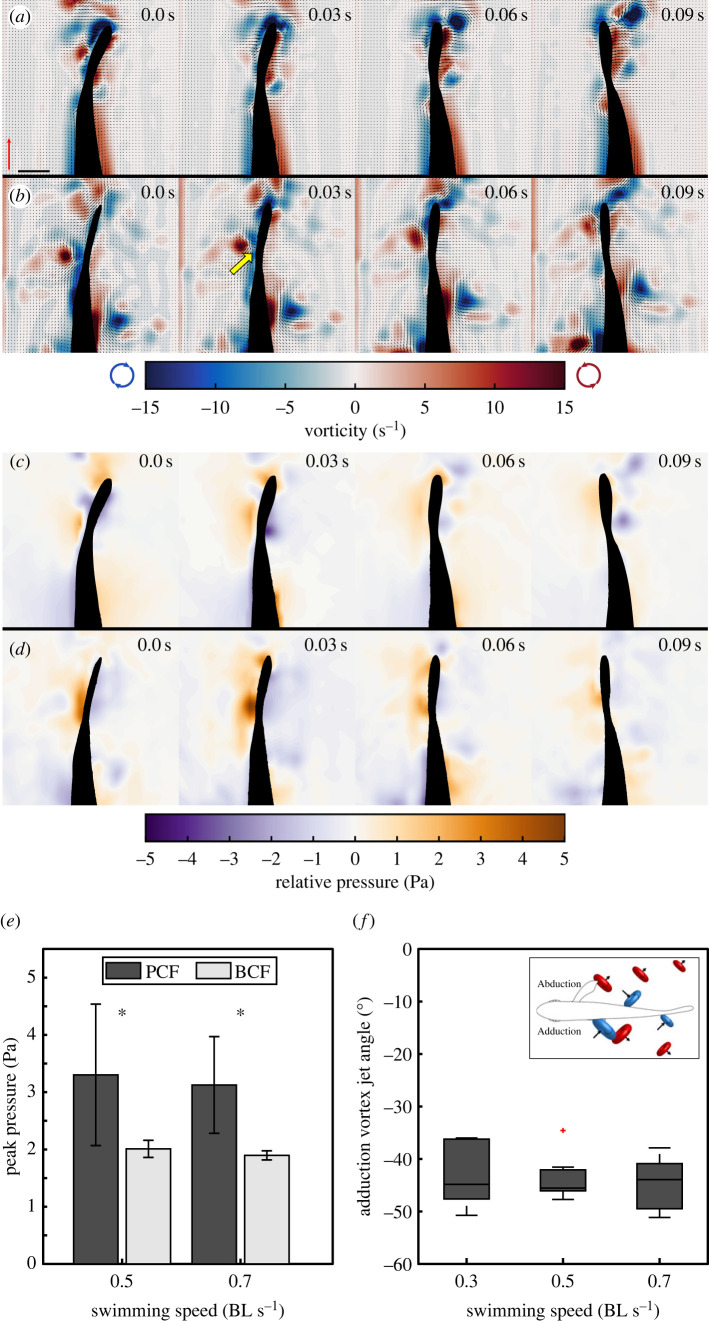
Instantaneous fluid vorticity and pressure fields. (*a*) Vorticity fields around the caudal fin of a fish swimming at 0.5 BL s^−1^, displaying BCF swimming. (*b*) Vorticity fields around the caudal fin of a fish swimming at 0.5 BL s^−1^, displaying PCF swimming. This enables the interception of the pectoral fin wake vortices by the tail. The yellow arrow indicates the moment a pectoral fin vortex interacts with the tail during PCF coordination. Two more incoming vortices are visible along the body and tail peduncle at *t* = 0.09 s. (*c*) Pressure fields around the caudal fin during BCF swimming. This generates a uniform increase in pressure along the tail. (*d*) Pressure fields around the caudal fin during PCF swimming. Here, vortex interception during PCF generates an area of localized high pressure. The red scale arrow indicates 0.5 m s^−1^ and the scale bar indicates 10 mm for all frames. Peak pressure measured at the caudal fin (*e*) was greater during PCF (*t*-test, *p* = 0.046). (*f*) Average vortex jet angle relative to the rostro-caudal axis (*n* = 9). No significant differences were observed across relative swimming speeds (ANOVA, *p* = 0.83). Negative angles indicate that vortex jets are oriented medially toward the caudal fin (inset).

PCF coordination shares some kinematics characteristics with asynchronous MPF and BCF swimming. However, the hydrodynamic outcomes of PCF gait are vastly different from either of the other two swimming modes (figure 1; electronic supplementary material, movies S1, S2 and S3). The fish displayed three distinct speed-dependent gaits: (1) asynchronous MPF locomotion at *U* ≤ 0.3 BL s^−1^, (2) PCF coordination at 0.3 BL s^−1^ ≤ *U* < 1.0 BL s^−^^1^, and (3) BCF at *U* > 1.0 BL s^−1^. MPF and PCF coordination showed some overlap at swimming speeds *U* = 0.3 BL s^−1^, though the fish showed some preference for PCF coordination (see electronic supplementary material, table S1). Contrary to MPF swimming, specimens displaying PCF coordination at 0.3 BL s^−1^ shed most of the pectoral fin wake medially. Note that during asynchronous MPF swimming, small flow features remained near the body of the animal. By contrast, a clear cut-off speed existed between 0.7 and 1.0 BL s^−1^, where the fish transitioned from PCF coordination to BCF. Experimentally derived fluid velocity and vorticity fields showed that during PCF coordination, a single vortex ring detached from the tip of each pectoral fin at the end of adduction (figure 1 and electronic supplementary material, figure S2). Because of the periodic nature of pectoral fin wake shedding and caudal fin movements, series of up to three vortices—two on one side of the fish and one on the opposite side—were continually generated (figures 1 and 2*b* and electronic supplementary material, S2, movie S2). The pectoral fin vortices were not shed in the free steam but instead were circulated along the body toward the superior lobe of the caudal fin (figures 1*b* and 2*b*, and electronic supplementary material, S2). The central jet of these vortices was oriented medially with an average angle of −43.8 ± 4.9°, thus carrying momentum in the direction of the tail (figure 2*f*). No significant differences in vortex jet angle were observed across relative swimming speeds (ANOVA, *p* = 0.83). Smaller individual vortices were produced at the tip of the pectoral fins at the end of fin abduction and were shed in the free stream with their central jet oriented laterally (electronic supplementary material, figure S2). They showed no physical interaction with the caudal fin (electronic supplementary material, figure S2*b*). Note that areas of elevated vorticity extend along the surface of the fish body (present at any speed) because fiction with the body wall slows the fluid, giving measurable vorticity. This is not the same as shed vortices which are coherent, rotating bodies of fluid in the free stream.

**Figure 3.**
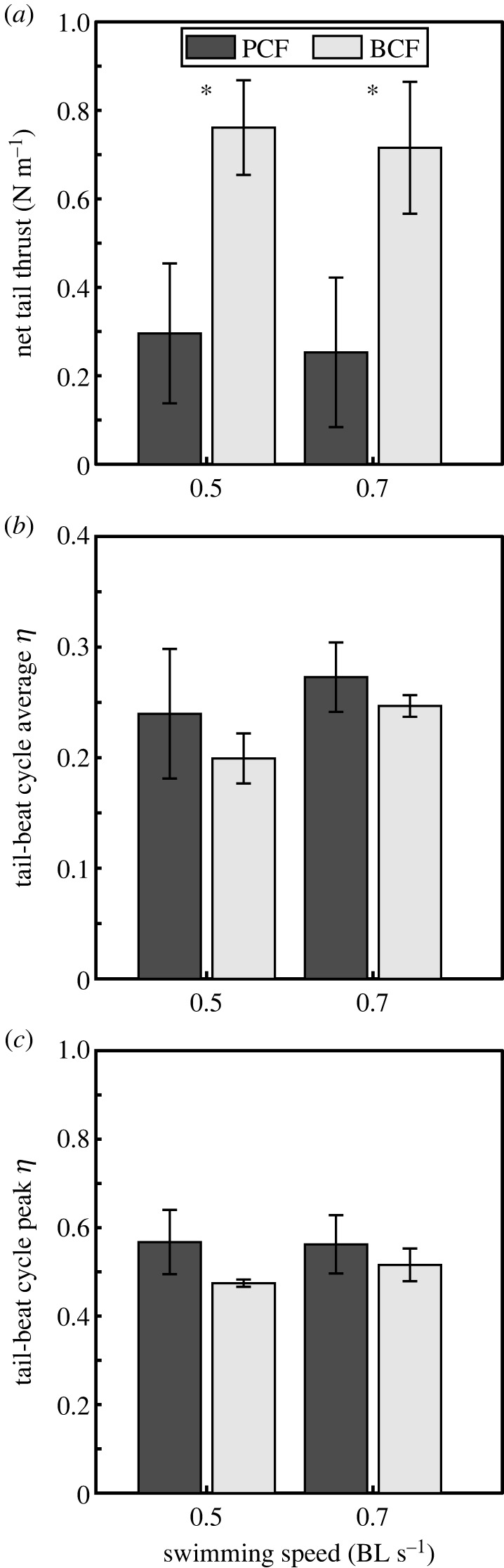
Fluid forces and hydrodynamic efficiency of the tail. (*a*) The net thrust produced by the tail over one tail beat cycle was less during PCF than during BCF at the same relative swimming speed (*t*-test, *p* = 0.014). Both the tail-beat cycle average (*b*) and peak (*c*) hydrodynamic efficiency ŋ were greater during PCF, though the limited number of occurrences of BCF at 0.5 and 0.7 BL s^−1^ (*n* = 2 and *n* = 3 respectively) was insufficient to make statistical inferences about the net efficiency gain of PCF coordination (*t*-tests, *p* = 0.42 and *p* = 0.23).

Fundamentally, the caudal fin operates under the same hydro-mechanical constraints during PCF coordination and BCF; a high-pressure area that resists movement forms along the flow-ward side during a lateral excursion (figures 1*e*,*f* and 2*c*,*d*). Asynchronous MPF swimming showed no identifiable pressure gradients at the tail (figure 1*d*). However, contrary to BCF and asynchronous MPF swimming, upstream pectoral fin vortices transferred momentum to the tail during PCF coordination. We recorded a momentary increase in pressure at the tail by 64% (*t*-test, *p* = 0.046) upon intercepting an upstream vortex (figure 2*c–e*) by comparing transient observations of fish displaying BCF at 0.5 and 0.7 BL s^−1^ (*n* = 2 and *n* = 3 respectively) with PCF coordination (*n* = 5 and *n* = 6, respectively). Positive pressure fields indicate reactive forces opposing motion [24]. The higher pressures observed at the caudal fin during PCF are the direct result of the medially oriented pectoral fin vortex jets doing work on the superior lobe of the caudal fin. This is supported in part by increased pressure at the tail during PCF coordination despite the narrower and slower lateral excursions of the caudal fin compared to BCF (ANOVA, *p* < 0.01) (see electronic supplementary material, figure S3). Tail beat amplitude during PCF coordination was, on average, 37.3% and 37.8% less than during BCF at 0.5 and 0.7 BL s^−1^, respectively. Because of these differences and the fact that during PCF swimming, a significant portion of the total net thrust was likely produced by the pectoral fins, thrust production by the tail during BCF and PCF swimming could not be readily compared to evaluate the potential contribution of upstream vortices to the net tail thrust. In fact, attempting to make this comparison shows that during PCF, the net tail thrust was, on average, only 38.9% and 35.4% of the thrust produced during BCF at 0.5 and 0.7 BL s^−1^, respectively (*t*-tests, *p* = 0.014 and *p* = 0.006; figure 3*a*). However, measurements of the hydrodynamic efficiency ŋ of the tail provide a better means to comparing PCF and BCF swimming. The limited use of BCF at 0.5 and 0.7 BL s^−1^ (*n* = 2 and *n* = 3, respectively) was insufficient to make statistical inferences about the net efficiency gain of PCF coordination. Nonetheless, on average pectoral fin wake recapture increased propulsive efficiency by 4.04% at 0.5 BL s^−1^ and 2.61% at 0.7 BL s^−1^, reaching *η* = 0.24 ± 0.06 and 0.27 ± 0.03, respectively (figure 3*b*,*c*).

### 3.2. Kinematics of pectoral–caudal fin coordination

**Figure 4.**
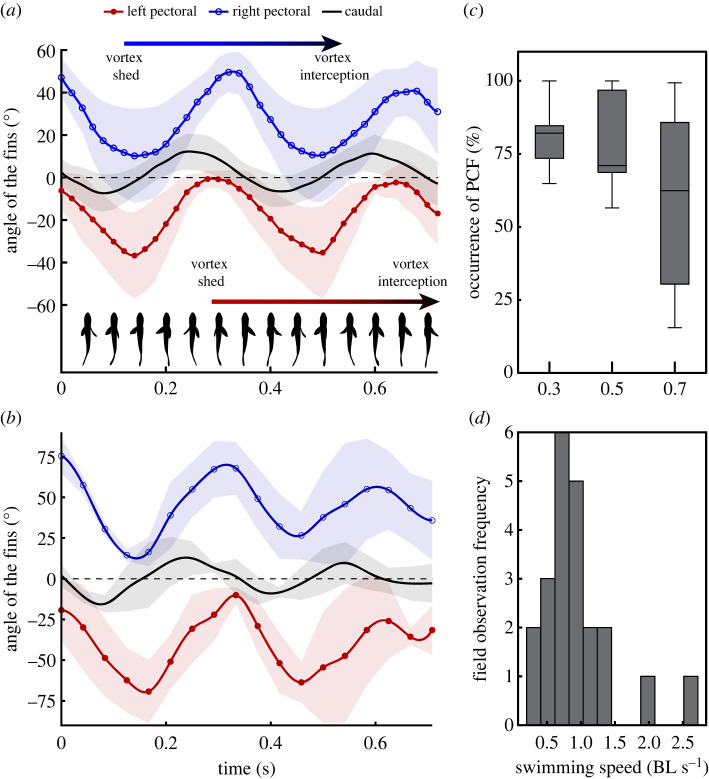
Kinematics and occurrence of PCF coordination. (*a*) Controlled experiments and (*b*) field observations show that PCF coordination is characterized by the temporal synchronization of the lateral displacement of both pectoral fins with the tail. Fin-beat kinematics is shown for a swimming speed *U* = 0.5 BL s^−1^. The caudal fin was coordinated with the pectoral fin vortex shedding pattern (horizontal arrows). (*c*) The fish maintained PCF for over 60% of the time during swimming experiments lasting 10 min. (*d*) Most of the fish observed in the wild swam between 0.5 and 1.0 BL s^−1^. All the fish displayed PCF within this range of relative swimming speeds. Only 4 fish displayed BCF locomotion for speeds > 1.0 BL s^−1^.

PCF coordination is characterized by the synchronized kinematics of two generally mutually exclusive swimming gaits: MPF swimming and BCF undulations. Controlled laboratory experiments revealed that mojarras temporally synchronized the movements of their pectoral fins with that of the caudal fin to swim steadily (figure 3*a*). The pectoral fins beat at a phase *ɸ* = 179.5 ± 22.2° regardless of swimming speed (*t*-test, *p* = 0.27; see electronic supplementary material), such that one fin is abducting while the other is adducting. The pectoral fins beat with a 78.8 ± 22.9° to 59.2 ± 25.2° phase shift relative to the caudal fin at 0.5 and 0.7 BL s^−1^, respectively (*t*-test, *p* = 0.038). When the tail moves to one side, the ipsilateral pectoral fin abducts, while the contralateral pectoral fin adducts. The wake vortices of the pectoral fins reach the caudal fin with a delay of precisely one full beat cycle (figure 4*a*). The tail is in phase with the upstream wake such that the tail-beat frequency and amplitude are driven by the shedding pattern of the pectoral fins (figure 4; electronic supplementary material, figures S4, S5). Consequently, the timing between vortex shedding and interception can be modulated simply by altering the phase shift between the pectoral fins and the tail (figure 4*a*).

*In situ* and *in vitro* data suggest PCF coordination is a pervasive swimming mode for relative swimming speeds ≤1.0 BL s^−1^ (figure 4*b*,*d*). Controlled experiments showed that the fish naturally selected PCF as their primary swimming mode at these speeds. On average, PCF was used 81.2 ± 11.8%, 78.7 ± 16.7% and 59.0 ± 32.0% of the time at 0.3, 0.5 and 0.7 BL s^−1^, respectively, although some fish displayed PCF for 100% of the time (figure 4*c*). In the wild, the fish selected areas where the flow was prone to maintaining PCF with equivalent swimming speeds of 0.5 to 1.0 BL s^−1^ (figure 4*d*). Up to 73% of the fish observed in the wild (*n* = 22) were swimming against tidal flows equivalent to these speeds (figure 4*d*). All these specimens displayed PCF. BCF swimming was exclusively observed for speeds >1.0 BL s^−1^ and only represented 18% of the total observations. The prevalence of PCF coordination in wild mojarras and its specific kinematics classify this behaviour as an active swimming mode rather than a transitory gait.

## 4. Discussion

From experimental and *in situ* observations of *E. argenteus* in tidal flows, we found a pervasive swimming gait involving the concurrent use of the pectoral fins and the caudal fin. PCF coordination emerges as a previously unrecognized swimming gait for recapturing energy and offers new avenues to explore the role of vortex interactions in aquatic propulsion to enhance efficiency and performance. While thrust-enhancing constructive flow interactions have been studied for fins arranged in line [10–12], we still need to elucidate the effects of the paired pectoral fins that can move large amounts of water rearward toward the caudal fin. Using experimental data, we show that mojarras can match the frequency and phase of the caudal fin with the wake pattern of the alternating pectoral fins on either side of the body. Upon interception, these vortices transfer energy to enhance the hydrodynamic efficiency of the tail, as seen through increased pressure and forces.

Experimental and theoretical works have demonstrated that the caudal fin can constructively use the wake shed by upstream median fins (i.e. dorsal and anal fins) by virtue of being coplanar, nearly contiguous, and phase-delayed [11,25]. However, experimental works indicate that the fluid momentum of the dorsal fin wake is about four times less than that produced by the pectoral fins [26], thus making the latter more suitable for moving large masses of water downstream for recapturing energy.

Compared to median fins, the laterally oriented, paired pectoral fins can produce more complex motions to vector forces for propulsion and manoeuvring [12,27,28]. Thus, pectoral fins have greater control in modulating their wake timing, direction, and strength. However, the geometry of the observed pectoral fin wake during PCF coordination differs substantially from the MPF swimmers studied to date [29] in two ways: (1) individual vortex rings generated at the end of fin adduction are circulated along the body rather than shed in the free stream (figure 1*b*; electronic supplementary material, figure S2) and (2) the central momentum jet of each vortex ring is oriented about 45° medially rather than laterally across all swimming speeds (figures 1*b* and 2*f*; electronic supplementary material, figure S2). By matching the frequency and phase of the caudal fin with the individual vortices shed on either side of the body by the alternating pectoral fins, the upstream wake momentum and energy are not lost laterally but rather recaptured by the tail (figures 1*b*,*e* and 2; electronic supplementary material, figure S2). This manifests by the convergence of fluid at the animal interface that causes a 64% increase of positive pressure fields along the caudal fin surface, compared with BCF swimming at the same speed (figure 2*c–e*). Positive pressure fields in the flow indicate reactive forces doing work on the body [24]. Given that pectoral fin vortices generate reactive forces on the flow-ward side of the tail during lateral excursions, acceleration reaction forces contribute to thrust. The efficiency of this gait in recapturing upstream wake energy depends on the successful synchronization of the wake patterns of the pectoral fins with lateral motions of the tail. Simple mechanical coupling via matching fin-beat frequencies is sufficient to maintain PCF coordination. However, stable and efficient PCF coordination may require sensory feedback mechanisms to inform the fish of the position and momentum of incoming upstream vortices to actuate the caudal fin accordingly. Individual vortices are circulated along the body towards the superior caudal fin lobe (electronic supplementary material, figure S2). This places these flow structures directly against the lateral line of the fish (electronic supplementary material, figure S2*c*). This organ provides sensory information relative to water motions and pressure gradients along the body [30] to potentially aid in performing fine adjustments of tail kinematics to optimize energy recapture.

Energy extraction from environmental flows, that have not been generated by the observed fish, has been consistently linked with the enhancement of the performance of swimming fish [31–34]. By recapturing vortex energy, fishes reduce muscle activity and, thus, adjust the energetic demand of the axial musculature to decrease the cost of transport [32,33]. Considering that the magnitude of the pressure gradient on either side of a propulsor is directly related to thrust production [35,36], pectoral fin wake interception emerges as a likely mechanism to enhance tail thrust and decrease cost of transport. Quantifying the potential gains of vortex recapture during PCF swimming is challenging. The tail has reduced kinematics compared with BCF swimming, thus invariably generating less overall thrust. The pectoral fins also partially contribute to the total thrust (excluding their effects downstream) and consequently lessen the overall contribution of the tail to total thrust. We found that the tail generates less thrust despite recapturing upstream vortices during PCF coordination. Reduced tail kinematics (i.e. lateral excursion velocity, amplitude) during BCF swimming generally achieves low performance but also exhibits low propulsive efficiency [11,37]. However, we found that by recapturing the upstream pectoral fin wake, the fish increased the propulsive efficiency of their tail by nearly 4% at 0.5 BL s^−1^. This gain results in a hydrodynamic efficiency of 0.24 ± 0.06, which exceeds that reported experimentally in another carangiform fish, bluefish (*Pomatomus saltatrix*) that uses BCF swimming at the same speed (ŋ = 0.18) [38]. The mean hydrodynamic efficiency for bluefish at speeds < 1.0 BL s^−1^ (speeds triggering PCF in mojarras) is ŋ = 0.19 ± 0.04 (*n* = 6). This is less than the hydrodynamic efficiency found with mojarras displaying the PCF mode at 0.5 and 0.7 BL s^−1^ (*η* = 0.24 ± 0.06 and 0.27 ± 0.03, respectively), and emphasizes the importance of vortex recapture by the tail to enhance efficiency.

There is mounting evidence that wake vortices directly enhance swimming performance [19,20]. Vortex rings provide a medium for swimming animals to push against (i.e. positive pressure fields), effectively acting as virtual walls [20]. The combined effects of vortex momentum and the energy stored in the central jet confer a propulsive advantage during PCF coordination by enhancing the transfer of propulsive forces despite reduced tail kinematics (compared to BCF at the same swimming speed). Previous works also proposed that upstream vorticity reinforces circulation around the caudal fin by adding energy to the tail's wake [11,39]. Because the fluid accelerates at the interface of interacting vortices, a high-velocity, low-pressure area forms along the propulsor, generating a suction force pulling the animal forward [40]. While this second mechanism alone would not explain the increase in pressure along the caudal fin during PCF coordination, we do not exclude its possible role in enhancing performance.

The potential energy stored in upstream flow structures offers an efficient means of harnessing energy that would otherwise be lost in the wake. The benefits of using the PCF mode at slow to moderate speeds, where BCF is deemed inefficient (reviewed in [5]), are amplified over long periods, such as a full tidal cycle. Given the relevance of these conditions to mojarras, even small changes in efficiency can lead to significant natural selection benefits [41]. It is, thus, crucial to consider the energy budget of swimming, particularly in the broader ecological context, as demonstrated by the prevalence of the PCF mode in wild mojarras (figure 4*d*). However, the immediate energetic benefits of PCF coordination over BCF still need to be clarified, as the two gaits only occur consistently at different swimming speeds. Thus, a direct cost of transport (COT) comparison between the gaits cannot be made. Likewise, a direct COT comparison with other species from the literature, which do not employ a PCF gait, is of little value due to differing size, shape and/or mass between the different species. However, we can compare the rate of change (i.e. slope) of COT for different species over the same relative swimming speeds to provide some insights. A strong negative slope in COT indicates a swim speed in which COT is rapidly declining and thus quickly becoming more efficient. A species with a weaker negative slope at the same relative swim speed indicates a more modest improvement in energy efficiency. In the silver mojarra (*E. argenteus*), we find that at 0.5 BL s^−1^ (a speed in which PCF is dominant), the negative slope of COT is approximately three times greater than for other species which do not exhibit PCF [42,43] (electronic supplementary material, figure S6). Beyond 1 BL s^−1^, when BCF swimming becomes the dominant mode in *E. argenteus*, the slopes of all species converge, suggesting a similar rate of change in COT. The fact that we find steeper slopes of COT at swimming speeds where mojarras employ PCF and vortex recapture implies an energetic efficiency advantage for this swimming mode. This may explain why we observed mojarras preferably selecting the PCF gait in nature where the habitats experience consistent flows of the magnitude in which mojarras swim using PCF in the laboratory.

But why is PCF abandoned at speeds >1 BL s^−1^? Previous studies on the bluegill showed a comparable gait transition from MPF to BCF swimming with increased recruitment of the axial musculature and the caudal fin near 1.0 BL s^−1^ [44]. Likely explanations for this arise from the increased power needed to actuate the pectoral fins with increasing swimming speeds. The high mechanical demand on the pectoral fins at speeds ≥1.0 BL s^−1^ marks potential physiomorphological and mechanical limits to how much the pectoral musculature can be recruited for propulsion. Therefore, fish eventually recruit the axial musculature, adapted to deliver more power to the tail [1]. Additionally, it may become increasingly difficulty to coordinate the ideal vortex interactions as swimming speed increases because the time from the moment a pectoral fin vortex sheds to when it needs to be intercepted shortens considerably (it is already only 0.31 ± 0.08 s at 0.5 BL s^−1^).

## 5. Conclusion

Understanding fish locomotion is fundamental to explaining the drivers of evolution, fitness and ecological interactions. The PCF coordination observed in *E. argentus* emerges as a previously unrecognized swimming gait for recapturing energy. Using experimental data, we show that fishes can synchronize their caudal fin with the wake pattern of the upstream alternating pectoral fins by matching the beat frequency and phase. In doing so, they transfer energy to the tail and receive minor gains in overall propulsive efficiency. Recent advances in the study of fish propulsion with robotic devices that measure power consumption [45–47] may help elucidate the energetic benefits of PCF relative to other swimming modes. While informing us about the ecology of fishes and the importance of maintaining swimming performance, incorporating these principles into bioinspired designs can serve as a basis for developing more efficient underwater vehicles. Lastly, more research is needed to evaluate the use of the PCF mode by other fish species and determine whether its particular functional and hydrodynamic characteristics extend the repertoire of the general classification of fish swimming modes.

## Data Availability

The fish swimming data and metadata files reported in this investigation have been deposited in the Zenodo digital repository: https://doi.org/10.5281/zenodo.824984 [48]. Custom Matlab scripts developed to compute kinematics, pressure fields and forces have also been made available. Supplementary material is available online [49].
